# Effect of intra-knee injection of autologous adipose stem cells or mesenchymal vascular components on short-term outcomes in patients with knee osteoarthritis: an updated meta-analysis of randomized controlled trials

**DOI:** 10.1186/s13075-023-03134-3

**Published:** 2023-08-10

**Authors:** Yang Yang, Zhibin Lan, Jiangbo Yan, Zhiqun Tang, Linghui Zhou, Dian Jin, Qunhua Jin

**Affiliations:** 1 Ningxia Medical University, The General Hospital of Ningxia Medical University, 804 Shengli South Street, Yinchuan, 750004 Ningxia Hui Autonomous Region China; 2 Institute of Medical Sciences, General Hospital of Ningxia Medical University, 804 Shengli South Street, Yinchuan, 750004 Ningxia China; 3 Ningxia Medical University, Lingwu People’s Hospital Affiliated to Ningxia Medical University, Lingwu, 750004 Ningxia China; 4 Ningxia Medical University, Yinchuan, 750004 Ningxia China

**Keywords:** Adipose-derived stem cells, Knee osteoarthritis, Stromal vascular fraction

## Abstract

**Objective:**

Assess the efficacy of single and multiple intra-articular injections of autologous adipose-derived stem cells (ASCs) and adipose-derived stromal vascular fraction (ADSVF) for the treatment of knee osteoarthritis (OA).

**Methods:**

We conducted a thorough and systematic search of several databases, including PubMed, Embase, Web of Science, Cochrane Library, and ClinicalTrials.gov, to identify relevant studies. The included studies were randomized controlled trials (RCTs) that involved single or multiple intra-articular injections of autologous ASCs or ADSVF for the treatment of patients with knee osteoarthritis, without any additional treatment, and compared to either placebo or hyaluronic acid.

**Results:**

A total of seven RCTs were analyzed in this study. The results of the meta-analysis show that compared to the control group, both single and multiple intra-articular injections of ASCs or ADSVF demonstrated superior pain relief in the short term (*Z* = 3.10; *P* < 0.0001 and *Z* = 4.66; *P* < 0.00001) and significantly improved function (*Z* = 2.61; *P* < 0.009 and *Z* = 2.80; *P* = 0.005). Furthermore, MRI assessment showed a significant improvement in cartilage condition compared to the control group. (*Z* = 8.14; *P* < 0.000001 and *Z* = 5.58; *P* < 0.00001).

**Conclusions:**

In conclusion, in osteoarthritis of the knee, single or multiple intra-articular injections of autologous ASCs or ADSVF have shown significant pain improvement and safety in the short term in the absence of adjuvant therapy. Significant improvements in cartilage status were also shown. A larger sample size of randomized controlled trials is needed for direct comparison of the difference in effect between single and multiple injections.

**Supplementary Information:**

The online version contains supplementary material available at 10.1186/s13075-023-03134-3.

## Introduction

Osteoarthritis (OA) of the knee is a prevalent degenerative joint disease that affects a staggering 350 million people worldwide [1, 2]. It is characterized by the gradual deterioration of articular cartilage, leading to pain, stiffness, and functional impairment. The economic burden of this condition is immense, with estimated indirect costs reaching as high as $13.2 billion annually [3].

Unfortunately, current treatment options for knee OA are limited and primarily focus on symptom relief rather than disease cure [4]. These treatments include medication for pain relief (steroidal or non-steroidal anti-inflammatory drugs and intra-articular injections of corticosteroids and hyaluronates), weight management, and the use of braces [5, 6]. However, these treatments eventually fail as OA progresses, and joint replacement surgery often becomes the last resort [5, 7].

In recent years, there has been a growing fascination with the potential use of stem cells as a therapeutic approach for treating knee osteoarthritis [8–10]. Among the various types of stem cells, mesenchymal stem cells (MSCs) have shown great promise in restoring damaged articular cartilage and slowing the progression of knee OA [11, 12]. Since autologous adipose tissue is easily available and abundantly sourced [13, 14], as a type of mesenchymal stem cells, adipose-derived mesenchymal stem cells (ASCs) and adipose-derived stromal vascular fraction (ADSVF) have been receiving increasing attention. ASCs, as a type of pluripotent stem cells, have the ability to self-renew and differentiate into multiple cell types. ADSVF refers to a cell population in adipose tissue, consisting of various cell types and extracellular matrix components, with the ability to promote angiogenesis and tissue repair. Although both have the potential to promote tissue repair and regeneration, they still exhibit differences in composition and function [8, 14, 15].

Previously, a meta-analysis including five studies investigated the efficacy of ASC and ADSVF treatments for osteoarthritis (OA) [16]. However, the findings were somewhat limited due to the small sample size and the limited number of studies included. Additionally, there was a lack of quantitative analysis on the number of injections of ADSVF and ASCs. Recently, two studies investigating ADVF and ASC were published that were not included in previous meta-analysis [17, 18]. Adding these studies will allow for subgroup analysis and more comprehensive evaluations. On this basis, two recently published papers were included in our meta-analysis, and subgroup analysis was performed for single or multiple different injection methods. This allows us to provide a more comprehensive evaluation of the effectiveness of different injection modalities in the treatment of knee OA and can provide some reference value for future therapeutic approaches.

## Methods

The study process was meticulously conducted in strict adherence to the Preferred Reporting Items for Systematic Reviews and Meta-analyses (PRISMA) reporting guideline [19]. Moreover, the program has been duly registered with PROSPERO (CRD42023418078), ensuring complete transparency and accountability.

### Data sources and searches

We conducted a thorough literature search using PubMed, Embase, Web of Science, Cochrane Library, and Clinical Trials.gov, covering publications up to April 20, 2023. Our search terms (Supplementary Table 1) included “adipose derived mesenchymal stem cell,” “stromal vascular fraction,” “knee,” “osteoarthritis,” and other synonyms. Additionally, we identified further references by reviewing the reference lists of relevant studies and reviews that were included.

### Selection of studies

After conducting a literature search, two researchers independently screened the title and abstract of each record. Studies were included in the current study if they met PICOS (patient, intervention, comparison, outcome, and study design) criteria (Supplementary Table 2) [20]. To ensure the highest level of data collection, only articles that unambiguously met the exclusion criteria were removed during the title and abstract screening process. The complete text of the remaining records was thoroughly reviewed, and only articles that met the inclusion criteria were included. In the event of any discrepancies between the two researchers, they were resolved through discussion or by consulting a third researcher.

The inclusion criteria for this study were as follows: (1) study topic: Efficacy of intra-articular injection of autologous adipose stem cells or interstitial vascular components in patients with knee osteoarthritis; (2) study design: clinical randomized controlled trial. The exclusion criteria were as follows: (1) irrelevant topics, lack of a control group or other cell-based therapies or control groups for PRP; (2) study designs such as review articles, case series, case reports, letters, conference abstracts, or reviews; (3) allogeneic cell therapy; (4) with other adjuvant therapeutic treatments such as platelet-rich plasma, corticosteroid, high tibial osteotomy, or cartilage repair procedures; (5) insufficient or inaccessible statistical information; (6) duplicate articles. The search was limited to articles published in English.

### Data extraction

The data extraction process for the study involved gathering the following information: (1) basic details such as the title, year of publication, and first author; (2) demographic characteristics including age, gender, and sample size; (3) the type of MSCs used and whether single or multiple injections were used for knee injections; (4) the visual analog scale (VAS) or Western Ontario and McMaster Universities Osteoarthritis Index (WOMAC) that could be utilized to evaluate the final outcome of relevant data; and (5) Whole-Organ Magnetic Resonance Imaging Score (WORMS) and magnetic resonance observation of cartilage repair tissue (MOCART) score to assess the final outcome of imaging. Two investigators independently conducted the data extraction, and any discrepancies were resolved through discussion or by seeking the opinion of a third investigator.

### Assessment of article quality

Randomized trials were assessed using the revised Rob2 (Version 2.0), considering sequence generation, allocation concealment, participant blinding, outcome assessment blinding, incomplete outcome data, and reporting bias [21]. Each aspect of the assessment was categorized as low risk of bias, high risk of bias, or unclear risk of bias and was performed independently by two investigators. Any discrepancies could be resolved by discussion or by seeking the opinion of a third investigator.

The quality of evidence for all outcomes was assessed by two researchers using the Recommended Assessment, Development, and Evaluation (GRADE) method (GRADE Pro, version 3.6). This assessment used five indicators, including risk of bias, inconsistency, indirectness, imprecision, and other considerations of bias, to assess each outcome. The level of evidence was categorized as high, moderate, low, or very low based on the likelihood that further research would affect confidence in the effect estimates.

### Outcomes and statistical analysis

The main statistical outcome measures included pain score (100-mm visual analog score [VAS]) and function score (total Western Ontario and McMaster University Osteoarthritis Index [WOMAC] score). Secondary outcome measures were MRI assessment (Whole-Organ Magnetic Resonance Imaging Score [WORMS], magnetic resonance observation of cartilage repair tissue [MOCART], and other cartilage improvements or structural changes) and safety (evaluated by procedure-related pain or swelling, adverse events [AEs], and serious AEs [SAEs]). In cases where data was missing, we will try to contact the author of the article in order to obtain the data. If this was unsuccessful, we used the formula outlined in the Cochrane Handbook for Systematic Reviews of intervention to calculate the missing values from other available data.

In our study, we evaluated the level of between-study heterogeneity by utilizing the *I*^2^ statistic. If the *I*^2^value is less than or equal to 50%, the heterogeneity between studies can be classified as low or moderate [22]. We employed a fixed effects model to combine effect values. However, if the *I*^2^ value exceeds 50%, the heterogeneity between studies is considered high, and we utilized a random effects model to combine effect values. The data analysis was carried out using Review Manager (RevMan) version 5.4 (Nordic Cochrane Center, Cochrane Collaboration).

## Results

### Characteristics of the included studies

**Fig. 1:**
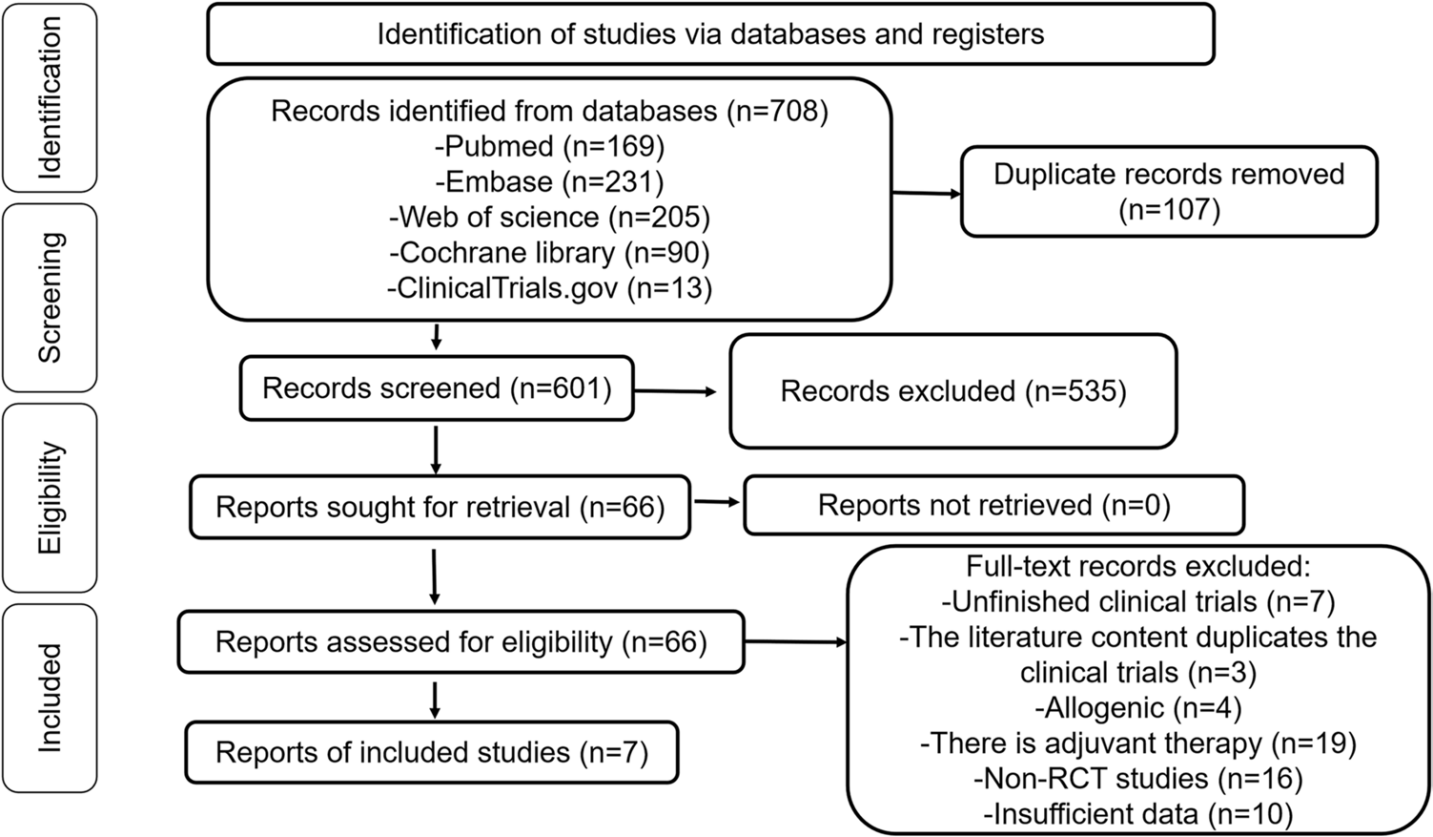
Selection process for systematic review

The process of selecting studies is shown in Fig. 1. After removing duplicates and irrelevant papers, we retrieved 708 records from different databases including Medline (accessed through PubMed), Embase, Web of Science, Cochrane Library, and ClinicalTrials.gov, and we assessed 66 published reports for eligibility to be included in the full-text assessment. Ultimately, seven RCT articles were considered suitable for inclusion in this meta-analysis (Table 1) [17, 18, 23–27].

**Table 1:** Characteristics of studies on osteoarthritis treatment using autologous adipose tissue

Characteristics	Zhang (2021) [23]	Zhang* (2021) [24]	Garza (2020) [25]	Freitag (2019) [26]	Hong (2019) [27]	Lu (2019) [17]	Lee (2019) [18]
Country	China	China	USA	Australia	China	China	South Korea
Journal	Stem Cell Res Ther	Biomed Res Int	Amj Sport Med	Regen Med	Int Orthop	Stem Cell Res Ther	Stem Cell Transl Med
Sample sizes, (*n*)							
Study	56	50	26	20	16	26	12
Control	70	50	13	10	16	26	12
Age, (years), mean ± SD							
Study	53.98 ± 13.69	50.83 ± 10.88	60.0 ± 9.8	57.4 ± 10.2	51.0 ± 6.0	55.0 ± 9.2	62.2 ± 6.5
Control	55.63 ± 12.18	52.87 ± 9.35	57.1 ± 9.1	51.5 ± 6.1	53.0 ± 11.0	59.6 ± 6.0	63.2 ± 4.2
Gender, male: female, (*n*)							
Study	14:42	18:29	15:11	11:09	3:13	3:23	3:09
Control	16:54	20:28	7:06	1:09	3:13	3:23	3:09
Body mass index, mean ± SD							
Study	23.73 ± 2.99	22.67 ± 3.68	28.2 ± 4.2	31.0 ± 5.6	26.3 ± 1.8	24.3 ± 3.0	25.3 ± 4.9
Control	23.86 ± 2.55	23.58 ± 4.19	27.1 ± 2.7	25.2 ± 3.4		24.3 ± 2.6	25.4 ± 3.0
Lower limb alignment	Mean varus 1.63 ± 2.21°for SVF; mean varus1.49 ± 2.12 for control group	NR	NR	< 5°varus or valgus for inclusion criteria	NR	Mean varus 1.4 for ASC; mean varus 0.4 for control group	NR
Follow-up (year, month)	1, 2, 3, 5 (year)	6, 12 (month)	1.5, 3, 6, 12 (month)	1, 3, 6, 12 (month)	1, 3, 6, 12 (month)	6, 12 (month)	3, 6 (month)
K-L grade	II, III	II, III	II, III	II, III	II, III	I, II, III	II, III, IV
Entity of cells	ADSVF	ADSVF	ADSVF	ASC	ADSVF	ASC	ASC
Control	HA	HA	Placebo (lactated Ringer solution)	Non-injection	HA	HA	Placebo (normal saline)
Delivery method	Direct IA	Direct IA	IA under US	IA under US	IA under arthroscopy	Direct IA	IA under US
Injection frequency	Thrice at 0, 1, and 2 months	Once	Once	Twice at 0, 6 months	Once	Twice at 0 and 3 weeks	Once
No. of cells (× 10^7^)	4.84 ± 1.61	NR	3 (high dose); 1.5 (low dose)	10	0.8	5	10
Adipose donor site	Abdomen	Abdomen	Abdomen	Abdomen	Abdomen	Abdomen	Abdomen
Rehabilitation	Non-weight bearing for 2 days and to undertake only light activity and avoid previously painful activities for the first 3 weeks after injection	NR	Minimal weight bearing for 2 days, with full range of motion. Light activity for the first 3 weeks after injection	Non-weight bearing for 4 weeks. Regarding range of motion and quadriceps activation exercises Non-weight bearing after the second injection at 6 months in two injection groups	Non-weight bearing for 1 days after operation and were discharged 2 days post operation with the same health propaganda	NR	Non-specific physical limitation was recommended from the day after the injection

*ADSVF* adipose-derived stromal vascular fraction; *ASC* adipose-derived stem cell; *HA* hyaluronic acid; *IA* intra-articular injection; *US* ultrasonography; *K-L grade*, Kellgren-Lawrence grade; *NR* not reported; Zhang*, in order to distinguish between two authors with the same last name and publication year, use “*” as a distinction

### Risk of bias

In terms of risk of bias, Supplementary Fig. 1 shows the results of the included studies evaluated. Almost all RCTs provide a relatively clear description of the randomization process. However, one RCT carries a high risk of bias due to a lack of participant blinding. The proportion of patients lost to follow-up was less than 20% in all studies, indicating a low risk of attrition bias, and the risk of bias for each item was expressed as a percentage of all trials, which illustrates the risk of bias ratio for each item.

### Outcomes of meta-analysis

#### Pain score at 6 months

**Fig. 2:**
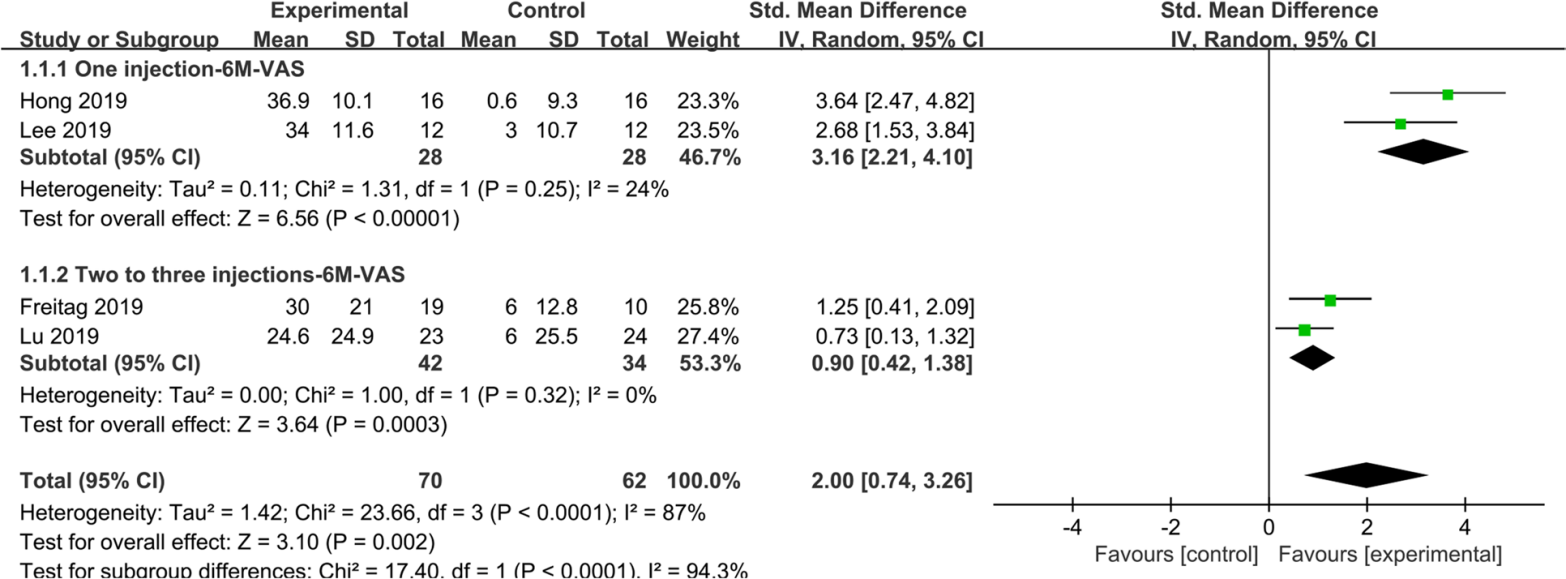
A total of 4 studies reported 100-mm VAS scores at 6 months, with mean improvement significantly higher in the overall study group than in the control group

A total of 4 studies reported 100-mm VAS scores at 6 months, with mean improvement significantly higher in the overall study group than in the control group (SMD: 2.00; 95% CI: 0.74–3.26; *I*^2^ = 87%; *Z* = 3.10; *P* < 0.0001) (Fig. 2). In addition, in a subgroup analysis of the study group, significantly greater improvements in 100-mm VAS were also observed in one injection groups (SMD: 3.16; 95% CI: 2.21–4.10; *I*^2^ = 24%; *Z* = 6.56; *P* < 0.00001) and two to three injections groups (SMD: 0.90; 95% CI: 0.42–1.38; *I*^2^ = 0%; *Z* = 3.64%; *P* < 0.0003) were more significant than the control group.

Similar results were obtained for subgroup analysis of ASCs and ADSVF. The improvement in the 100-mm VAS score at 6 month was significantly higher in the ADSVF (Supplementary Fig. 2).

#### Pain score at 12 months

**Fig. 3:**
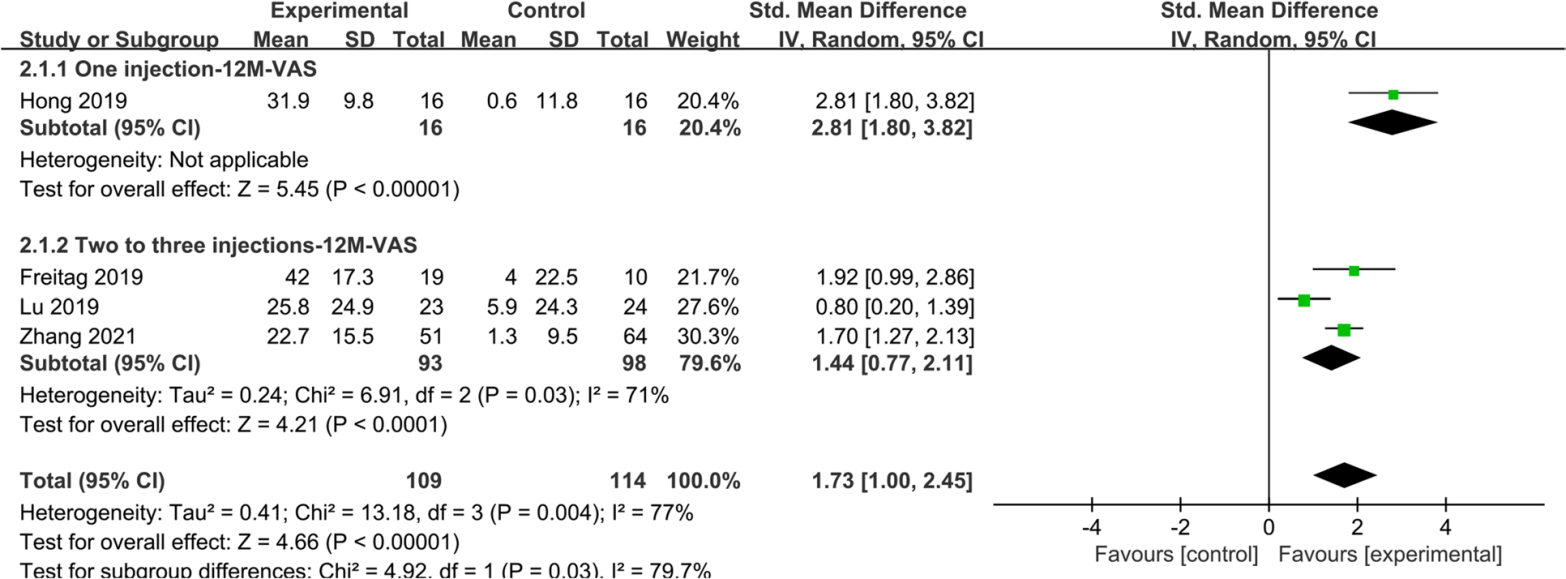
A total of 4 studies reported 100-mm VAS scores at 12 months, with mean improvement significantly higher in the overall study group than in the control group

A total of 4 studies reported 100-mm VAS scores at 12 months, with mean improvement significantly higher in the overall study group than in the control group (SMD: 1.73; 95% CI: 1.00–2.45; *I*^2^ = 77%; *Z* = 4.66; *P* < 0.00001) (Fig. 3). In the subgroup analysis of the study group, improvements in 100 mm VAS were observed in both the one injection group (SMD, 2.81; 95% CI: 2.81–3.82; *Z* = 5.45; *P* < 0 0.00001) and in the two to three injections group (SMD: 1.44; 95% CI: 0.77–2.11; *I*^2^ = 71%; *Z* = 4.21; *P* < 0.0001) compared to the control group.

The subgroup analysis of ASCs and ADSVF showed that the VAS scores at 12 months may be better than those of the control group (Supplementary Fig. 3).

#### Total WOMAC score at 6 months

**Fig. 4:**
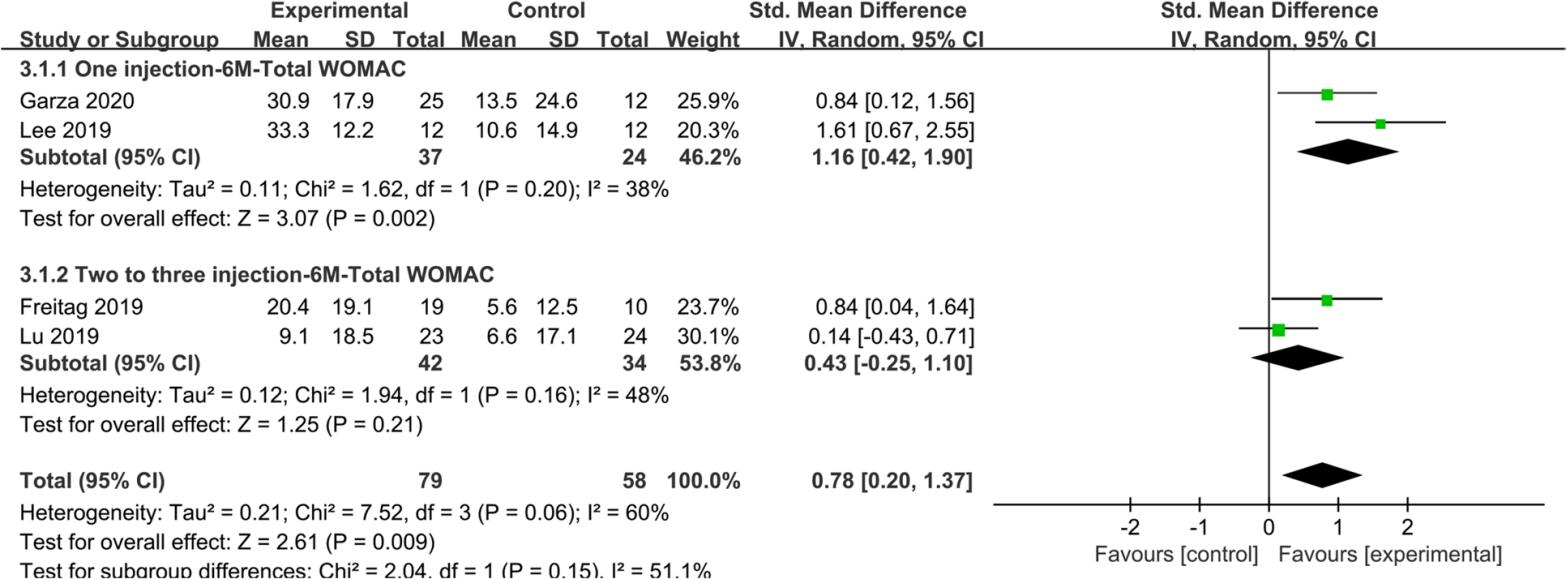
Four studies reported total WOMAC scores at 6 months, with the experimental group improving significantly more than the control group

Four studies reported total WOMAC scores at 6 months, with the experimental group improving significantly more than the control group (SMD: 0.78; 95% CI: 0.20–1.37; *I*^2^ = 60%; *Z* = 2.61; *P* = 0.009) (Fig. 4). In addition, in a subgroup analysis, the one injection group (SMD: 1.16; 95% CI: 0.42–1.90; *I*^2^ = 38%; *Z* = 3.07; *P* = 0.002) was significantly different from the control group at 6 months. In contrast, the results were reversed in the two to three injection group (SMD: 0.43; 95% CI: − 0.25–1.10; *I*^2^ = 48%; *Z* = 1.25; *P* = 0.21), which was not significantly different from the control group at 6 months.

The subgroup analysis of ASCs and ADSVF showed that ADSVF group had a significantly better total WOMAC score than the control group at 6 months; however, the ASC group results showed no significant difference (Supplementary Fig. 4).

#### Total WOMAC score at 12 months

**Fig. 5:**
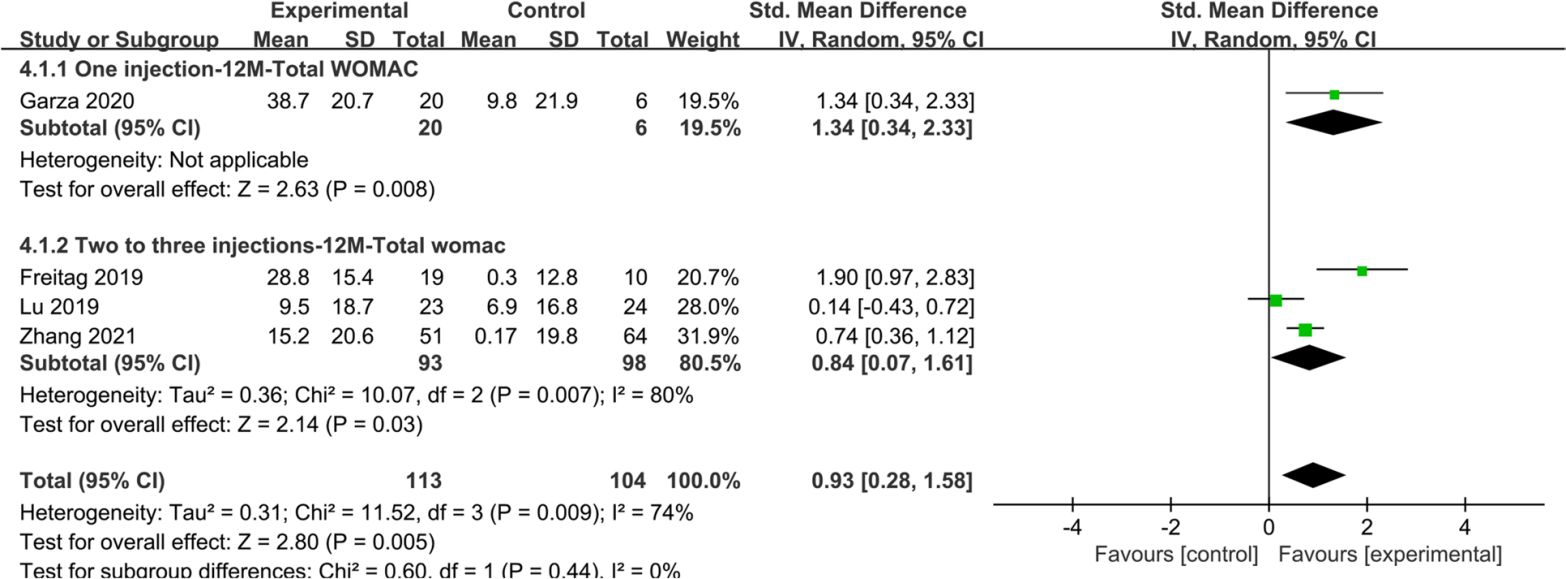
Four studies reported total WOMAC scores at 12 months, with the experimental group improving significantly more than the control group

Four studies reported total WOMAC scores at 12 months, with the experimental group improving significantly more than the control group (SMD: 0.93; 95% CI: 0.28–1.58; *I*^2^ = 74%; *Z* = 2.80; *P* = 0.005) (Fig. 5). In the subgroup analysis, improvements in total WOMAC score were observed in both the one injection group (SMD: 1.34; 95% CI: 0.34–2.33; *Z* = 2.63; *P* = 0 0.008) and two to three injections group (SMD: 0.84; 95% CI: 0.07–1.61; *I*^2^ = 80%; *Z* = 2.14; *P* = 0.03) compared to the control group.

The subgroup analysis of ASCs and ADSVF showed that the ADSVF group had a better total WOMAC score than the control group at 12 months, and the ASC group results showed no significant difference (Supplementary Fig. 5).

#### WORMS of the ADSVF injection group

Two studies reported total WORMS at 6 and 12 months, with the experimental group improving significantly more than the control group (SMD: 24.11; 95% CI: 18.30–29.92; *I*^2^ = 51%; *Z* = 8.14; *P* < 0.00001) (Supplementary Fig. 6). In the subgroup analysis, the WORMS was significantly higher in the 6-month group (SMD: 19.29; 95% CI: 14.23–24.36; *I*^2^ = 0%; *Z* = 7.47; *P* < 0.00001) and in the 12-month group (SMD: 27.56; 95% CI: 22.68–32.44; *I*^2^ = 0%; *Z* = 11.07; *P* < 0.00001) were significantly different than the control group in the studies of injections of ADSVF.

#### MOCART score at 6 and 12 months

Two studies reported total WORMS at 6 and 12 months, with the experimental group improving significantly more than the control group (SMD: 11.82; 95% CI: 7.86–15.78; *I*^2^ = 0%; *Z* = 5.58; *P* < 0.00001) (Supplementary Fig. 7).

#### Other MRI outcomes

Due to the heterogeneity of assessment methods and the limited number of studies, it was not possible to conduct a meta-analysis on other indicators of cartilage or structural. In conclusion, out of the 7 studies we analyzed, 4 studies demonstrated a significant improvement in cartilage status in the ASC or ADSVF group compared to the control group [17, 18, 24, 27], while 2 studies showed no significant change [25, 26]. Additionally, a long-term study spanning 5 years reported no significant change [23]. The specifics of the MRI assessment are presented in Table 2.

**Table 2:** MRI evaluation of cartilage regeneration in osteoarthritis

Lead author (year) Cell type	MRI Protocol	Assessment	F/U (mo, y)	Cartilage pathology		Overall results of cell therapy
				Study	Control	
Zhang (2021) ADSVF	PDFS	Total cartilage volume (mm^3^)	Baseline	16,467.89 ± 2739.13	15,718.20 ± 2071.90	The defect size increased more in the control group. The SVF group had fewer patients experiencing progression
			5 y	15,121.11 ± 3174.45	13,473.30 ± 2489.59	
		Full-thickness defect	5 y	Decrease: 3 (5.9%) No change: 44 (86.3%)	Decrease: 0 (0%) No change: 52 (81.3%)	
Zhang* (2021) ADSVF	PDFS and 3D-FS-SPGR	WORMS	Baseline	64.28 ± 13.90	63.99 ± 13.38	The study group showed significant improvement at 6 and 12 months, while the control group experienced significant deterioration at the same time points
			6 mo	53.88 ± 12.61	72.16 ± 13.74	
			12 mo	48.17 ± 11.40	74.97 ± 12.80	
		MOCART	6 mo	50.00(12.60)	21.77 (13.34)	
			12 mo	59.81 (12.59)	18.24 (9.48)	
Garza (2020) ADSVF	PDFS	Cartilage thickness (change)	6 mo	− 0.2 mm	10.5 mm	Modified disease progression No difference No change after treatment
			12 mo	− 0.1 mm	10.8 mm	
		Outerbridge grade (change)	6 mo	0	0	
			12 mo	0	0	
Freitag (2019) ASC	PDFS	MOAKS	12 mo	Improved: 1/19 (5.3%) No change: 14/19 (73.7%) Progression: 4/19 (21.0%)	Improved: 0/9 (0%) No change: 3/9 (33%) Progression:6/9 (67%)	Tended to be better in the ASC group than in the control group, but not significant
Hong (2019) ADSVF	PDFS	WORMS	Baseline	71.31 ± 24.2	69.81 ± 18.05	The study group showed significant improvement at 6 and 12 months, while the control group experienced significant deterioration at the same time points
			6 mo	59.93 ± 24.89	82.62 ± 12.66	
			12 mo	55.87 ± 21.95	85.31 ± 14.65	
		MOCART	6 mo	54.06 (11.58)	19.38 (9.64)	
			12 mo	62.81 (8.16)	19.06 (7.79)	
Lu (2019) ASC	PDFS	Cartilage volume (change)	6 mo	Significantly increased in the right knee	Significantly decreased in the left tibia	Significant difference in the left tibia and right femur (ASC treatment was better than HA)
			12 mo	Significantly increased in both femurs	No significant change	Significant difference in both femurs ASC treatment was better than HA
Lee (2019) ASC	PDFS	Cartilage defect size (mm2)	Baseline	312.47 ± 270.97	320.02 ± 273.02	No significant change in the ASC group; significant increase in defect size in the control group. ASC treatment better than placebo
			6 mo	314.86 ± 267.33	355.61 ± 258.54	
			Gap	+ 2.4 ± 14.5	+ 35.6 ± 58.8	

*ADSVF* adipose-derived stromal vascular fraction; *ASC* adipose-derived stem cell; *F/U* follow-up; *HA* hyaluronic acid; *MOCART* magnetic resonance observation of cartilage repair tissue; *MOAKS* MRI Osteoarthritis Knee Score; *MRI* magnetic resonance imaging; *PDFS* proton density fat saturated; *3D-FS-SPGR* three-dimensional fat-suppressed spoiled gradient recalled echo sequence; *WORMS* Whole-Organ Magnetic Resonance Imaging Score; *mo* month; *y* year

### Adverse reactions

In all of the studies analyzed, the occurrence of knee pain or swelling related to surgery was found to be 46% in both the treatment and control groups. The combined hazard ratio estimate was 1.04 (95% CI, 0.82–1.31; *I*^2^ = 41%; *Z* = 0.30), indicating that there was no statistically significant difference between the two groups (*P* = 0.77) (Supplementary Fig. 8). Supplementary Table 3 provides further details on the adverse events reported in the studies, with no reports of serious adverse events associated with ASC or ADSVF treatment.

### Subgroup analysis and meta regression results

Supplementary Table 4 presents the summary results of the subgroup analyses. Supplementary Table 5 summarizes the meta-regression analysis, revealing no significant sources of heterogeneity.

### Quality of the evidence and recommendation strengths

The evidence quality for all the findings was either moderate or low, with no instances of very low evidence levels. As a result, we concur that the overall quality of the evidence is moderate, indicating that the actual effects may be comparable to the estimated effects. The results indicate that both single and multiple intra-knee injections of ADSVF or ASC may have a dependable short-term impact on knee osteoarthritis (Supplementary tables 6 and 7).

## Discussion

The results of this meta-analysis showed that (1) both single and multiple injections of ASCs or ADSVF improved pain and function in patients with OA, and (2) the subgroup analysis revealed that both single and multiple injections were found to significantly improve pain relief in patients suffering from knee osteoarthritis when compared to controls. However, differences in functional efficacy exist, and further large sample long-term follow-up studies are needed for direct comparison; (3) there was a significant improvement in cartilage status of osteoarthritic knee joints in the ASC or ADSVF groups; (4) and there was no difference in surgery-related pain or swelling between the ASCs or ADSVF groups and the control group.

Specifically, the mean VAS improvement ranged from 24.6 to 36.9 at 6 months and 22.7 to 42.00 at 12 months in the single injection and two to three injection groups, while the mean VAS improvement ranged from 0.6 to 6.0 at 6 months and 0.6 to 5.9 at 12 months in the control group. After comparing treatment plans, Freitag et al. [26] demonstrated that both single intra-articular injection of ASCs and two injections at 6-month intervals improved OA pain and function.

This meta-analysis showed that there was no significant difference in total WOMAC between the two to three injection groups compared to the control group at 6 months. Lu et al. [17] showed that there was no statistically significant difference in the improvement of total WOMAC scores between ASCs and HA at 6 and 12 months. However, the study also noted a trend toward better cure rates after injection of ASCs than in the control group. In addition, the results of the meta-analysis indicated that both single and two to three injections significantly improved total WOMAC at 12 months compared to placebo or HA injections. Emadedin et al. [28] conducted long-term follow-up of the same cohort demonstrated that the dosage of bone marrow MSCs was both safe and therapeutically beneficial. However, therapeutic improvement declined between 12 and 30 months in all individuals, suggesting the need for subsequent dosing to prolong efficacy [29]. It is therefore reasonable to believe that multiple frequent injections are warranted to ensure long-term efficacy.

The role of ADSVF in cartilage regeneration is also reflected in this piecewise meta-analysis. Of the seven included papers, it was beneficial that two papers [24, 27] evaluated cartilage changes using the same methodology, thus allowing us to perform a quantitative meta-analysis. The results of this review showed that ADSVF injection significantly improved WORMS scores at 6 and 12 months. The MOCART scores in these two studies similarly reflect this view. Zhang et al. [24] found significant defect filling and cartilage repair in the knee joint after receiving ADSVF, with a higher increase in grade 2 OA than grade 3 OA after treatment. Many clinical studies [29, 30] have shown the potential efficacy of MSCs, including ASCs and ADSVF, for cartilage regeneration in patients with knee OA, which is consistent with our results. Notably, most current studies have yielded short-term results that MSCs, including ASCs or ADSVF, are effective in alleviating OA cartilage degeneration, but the efficacy of these therapeutic modalities for OA cartilage regeneration remains controversial [8, 30–33].

In terms of safety, we found that adipose-derived MSCs for osteoarthritis had fewer adverse effects, mainly including local pain and swelling, but most of these reactions were mild and transient and did not require special treatment. Our meta-analysis showed no difference in surgery-related pain or swelling between the ASCs or ADSVF groups and the control group, which is consistent with a recent meta-analysis [13, 16, 33, 34]. In addition, we also noted some potential safety issues in some studies, such as the source and quality control of stem cells, injection dose, and modality, which need to be further studied and resolved. In conclusion, adipose-derived MSCs have high safety in the treatment of osteoarthritis.

ASCs and ADSVF are the commonly used types of adipose tissue MSCs treatment. Theoretically, there is a relationship between the efficacy of MSCs treatment for osteoarthritis and the number of intra-articular injections. However, there is no literature to suggest that the greater the number of intra-articular MSCs injections, the better the efficacy. In contrast, Hong et al. [27] showed that a single intra-articular injection of MSCs can also significantly improve pain and function in patients with osteoarthritis. In this meta-analysis, the only study comparing the two approaches showed that two ASCs injections were superior to a single injection in terms of early stabilization of articular cartilage degeneration. Although our subgroup analysis reached similar conclusions, these studies do not allow us to draw conclusions about the efficacy between single intra-articular MSCs injections and multiple injections because of the inherent statistical limitations of indirect comparisons. Therefore, the current studies show limited evidence of clinical efficacy of ASCs and ADSVF. A large number of direct comparative studies are needed to provide stronger evidence in the future. Provide reasonable dosing and injection modalities to ensure the safety and efficacy of MSCs therapy for OA.

This article has some limitations that need to be addressed. Firstly, the literature included on uniform assessment criteria for MRI is not extensive enough, which may affect the accuracy of the findings. Secondly, the evidence supporting subgroup analysis may not be sufficient, which may limit the generalizability of the results. Thirdly, the different sample sizes of each study may introduce bias to the final results, which may affect the reliability of the conclusions. Fourth, despite strict inclusion criteria, heterogeneity in injection dose, injection concentration, rehabilitation modality, and control group may create a potential risk of bias. Moreover, the number of long-term follow-up studies is insufficient, which may interfere with studies of long-term efficacy and limit the practical implications of the research.

## Conclusions

In osteoarthritis of the knee, single or multiple intra-articular injections of autologous ASCs or ADSVF have shown significant pain improvement and safety in the short term in the absence of adjuvant therapy. Significant improvements in cartilage status were also shown on MRI. A larger sample size of randomized controlled trials is needed for direct comparison of the difference in effect between single and multiple injections.

### Supplementary Information

**Additional file 1: Supplementary table 1.** Details of search strategy. **Supplementary table 2.** PICOS. **Supplementary table 3.** Adverse and serious adverse events in the included studies. **Supplementary table 4.** Results of subgroup analysis. **Supplementary table 5.** Meta-regression results. **Supplementary table 6.** Grade evidence profile1. **Supplementary table 7.** Grade evidence profile2

**Additional file 2: Supplemental Fig. 1.** Risk of bias assessment of the included studies, involving a risk of bias graph and summary.

**Additional file 3: Supplemental Fig. 2.** Meta-analysis of VAS scores at 6 months for the ADSVF and ASCs subgroups. **Supplemental Fig. 3.** Meta-analysis of VAS scores at 12 months for the ADSVF and ASCs subgroups. **Supplemental Fig. 4.** Meta-analysis of total WOMAC scores at 6 months for the ADSVF AND ASCs subgroups. **Supplemental Fig. 5.** Meta-analysis of total WOMAC scores at 12 months for the ADSVF AND ASCs subgroups. **Supplemental Fig. 6.** Meta-analysis of total WORMS in ADSVF injection group. **Supplemental Fig. 7.** Meta-analysis of MOCART between 6 and 12 months. **Supplemental Fig. 8.** Meta-analysis of adverse reactions

## Data Availability

The datasets used and analyzed during the current study are available from the corresponding author on reasonable request.

## Abbreviations

ASCs: Adipose-derived stem cells
ADSVF: Adipose-derived stromal vascular fraction
JSW: Joint space width
OA: Osteoarthritis
RCTs: Randomized controlled trials
MSCs: Mesenchymal stem cells
VAS: Visual analog scale
WOMAC: Western Ontario and McMaster Universities Osteoarthritis Index
WORMS: Whole-Organ Magnetic Resonance Imaging Score
MOCART: Magnetic resonance observation of cartilage repair tissue
AEs: Adverse events
SAEs: Serious AEs
HA: Hyaluronic acid
IA: Intra-articular injection
US: Ultrasonography
