# Supplementary material for: Effect of intra-knee injection of autologous adipose stem cells or mesenchymal vascular components on short-term outcomes in patients with knee osteoarthritis: an updated meta-analysis of randomized controlled trials
Source: Arthritis Res Ther. 2023 Aug 10;25:147. doi: 10.1186/s13075-023-03134-3 (PMC10413774; doi:10.1186/s13075-023-03134-3)
Supplement: Supplementary file 1 — Additional file 1: Supplementary table 1. Details of search strategy. Supplementary table 2. PICOS. Supplementary table 3. Adverse and serious adverse events in the included studies. Supplementary table 4. Results of subgroup analysis. Supplementary table 5. Meta-regression results. Supplementary table 6. Grade evidence profile1. Supplementary table 7. Grade evidence profile2 [file 13075_2023_3134_MOESM1_ESM.doc]

**Supplementary Table 1**. Details of search strategy

| Database | Search strategy |
| --- | --- |
| PubMed | 1. (adipose derived mesenchy-mal stem cell[MeSH Terms]) OR (adipose derived culture expanded mesen-chymal stem cell[Title/Abstract]) OR (adipose derived stem cell[Title/Abstract]) OR (stromal vascular fraction[Title/Abstract]) OR (adipose tissue stromal vascular fraction[Title/Abstract]) 2. (knee[MeSH Terms]) OR (knee joint[Title/Abstract]) OR (patellofemoral joint[Title/Abstract]) OR (knee-joint[Title/Abstract]) OR (articulatio genus[Title/Abstract]) OR (gonitis[Title/Abstract]) 3. (osteoarthritis[MeSH Terms]) OR (arthrophlogosis[Title/Abstract]) OR (arthritis[Title/Abstract]) OR antarthritic([Title/Abstract]) OR (osteoarthritic[Title/Abstract]) OR (arthrophlogosis[Title/Abstract]) 4. #1 AND #2 AND #3 |
| Embase | 1. "adipose derived mesenchy-mal stem cell"/exp 2. "adipose derived culture expanded mesen-chymal stem cell"/exp 3. "adipose derived stem cell":ab,ti OR "stromal vascular fraction":ab,ti OR "adipose tissue stromal vascular fraction":ab,ti 4. #1 OR #2 OR #3 5. "knee"/exp 6. "knee joint"/exp 7. "knee-joint":ab,ti OR "articulatio genus":ab,ti OR "gonitis":ab,ti 8. #5 OR #6 OR #7 9. "osteoarthritis"/exp 10. "arthritis"/exp 11. "arthrophlogosis":ab,ti OR "antarthritic":ab,ti OR "osteoarthritic*":ab,ti 12. #9 OR #10 OR #11 13. #4 AND #8 AND #12 AND [English] |
| Web of science | 1. (AB=(adipose derived mesenchy-mal stem cell) OR AB=(adipose derived culture expanded mesen-chymal stem cell) OR AB=(adipose derived stem cell) OR AB=(stromal vascular fraction) OR AB=(adipose tissue stromal vascular fraction) ) 2. AB=(knee) OR AB=(knee joint) OR AB=(knee-joint) OR AB=(patellofemoral joint) OR AB=(articulatio genus) OR AB=(gonitis) 3. AB=(osteoarthritis) OR AB=(arthrophlogosis) OR AB=(arthritis) OR AB=(osteoarthritic) OR AB=(arthrophlogosis) OR AB=(antarthritic) 4. #1 AND #2 AND #3 AND #4 |
| Cochrane library | adipose derived mesenchy-mal stem cell* or adipose derived culture expanded mesen-chymal stem cell or adipose derived stem cell or stromal vascular fraction or adipose tissue stromal vascular fraction  **And**: knee or knee joint or knee-joint or patellofemora joint or articulatio genus or gonitis  **And**: osteoarthritis or arthrophlogosis or arthritis or arthrophlogosis or osteoarthritic |

**ClinicalTrials.gov**

Condition or disease: adipose derived mesenchy-mal stem cell or adipose derived stem cell

Other terms: osteoarthritis or osteoarthritic

Study Results: Studies with Results

**Supplementary Table 2**. PICOS

| PICOS | Inclusion Criteria | Exclusion Criteria |
| --- | --- | --- |
| Population | Patients with knee osteoarthritis | Animal study or in vitro study |
| Intervention | Intra-articular single or  multi-frequency injection of  autologous stromal vascular  MSCs derived from adipose  tissue or culture-expanded  MSCs. | Adjuvant treatments such as  platelet-rich plasma, cartilage  repair procedures,  Corticosteroid or  high-tibial osteotomy  Allogenic cell therapy  Biologic adjuvants such as fibrin |
| Comparison | Placebo or control group | Other cell-based therapy or platelet-rich plasma |
| Outcome | Patient-reported outcome measure (function and pain);  magnetic resonance imaging; adverse effect |  |
| Study design (level of evidence) | 1 | 2, 3, 4, or 5 |

**Supplementary Table 3**. Adverse and serious adverse events in the included studies

| Lead Author (Year) | Cell Type | Adverse Events | Serious Adverse Events |
| --- | --- | --- | --- |
| Zhang（2021） | ADSVF | There were no adverse reactions during postoperative follow-up in either group. | None |
| Zhang*（2021） | ADSVF | 21 patients (22.11%) had minor adverse events（AE）.  All AE were pain and swelling of the knee. | None |
| Garza(2020) | ADSVF | 3 patients (11.5%) had minor adverse events.  1 with knee swelling (negative aspiration), 2 patients  cultures were positive for bacteria but patients remained asymptomatic 6-12 month: no adverse events | None |
| Freitag(2019) | ASC | None: 15%.  Mild: 55%.  Moderate: 20%.  Severe: 10% patients had pain and swelling for 4 weeks and observed  an effect on their usual daily activity. | None |
| Hong(2019) | ADSVF | 4 patients (25%) had abdominal pain.  6 patients (37.5%) had pain and swelling in knee joints.  All of these adverse events were resolved by pain medication. | None |
| Lu(2019) | ASC | Similar proportion between the ASC (73.1%) and the HA (55.9%) groups.  The most common symptoms were pain and swelling of the injection site.  Spontaneous relief within 7 days without special treatment. | 1 for the HA group  (infection 2 months  after injection) |
| Lee(2019) | ASC | Adverse events occurred in 10 (83%) patients in the MSC group and 7 (58%) patients in the control group.  8 patients (66.75%) in the ASC group had treatment-related adverse events, including arthralgia in 6 patients and joint effusion in 2 patients.  All of these events were resolved by pain medication. | None |

**Supplementary Table 4**. Results of subgroup analysis.

| Subgroup | No. of studies | SMD(95%CI) | I^2^ (%) | P of heterogeneity |
| --- | --- | --- | --- | --- |
| 1.VAS score at 6 month |  |  |  |  |
| ASC | 1 | 3.64(2.47, 4.82) | / | < 0.00001 |
| ADSVF | 3 | 1.45(0.43, 2.47) | 77 | 0.005 |
| 2.VAS at 12 month |  |  |  |  |
| ASC | 2 | 2.16(1.08, 3.32) | 75 | 0.05 |
| ADSVF | 2 | 1.30(0.20, 2.40) | 75 | 0.05 |
| 3.WOMAC score at 6 month |  |  |  |  |
| ASC | 1 | 0.84(0.12, 1.56) | / | < 0.02 |
| ADSVF | 3 | 0.80(-0.04, 1.63) | 72 | < 0.03 |
| 4.WOMAC score at 12 month |  |  |  |  |
| ASC | 2 | 0.85(0.40, 1.31) | 17 | 0.27 |
| ADSVF | 2 | 0.98(-0.74, 2.70) | 90 | 0.002 |
| 5.WORMS score after ADSVF injection |  |  |  |  |
| 6 month | 3 | 19.29(14.23, 24.36) | 0 | 0.47 |
| 12 month | 2 | 27.56(22.68, 32.44) | 0 | 0.62 |
| 6.VAS score at 6 month |  |  |  |  |
| One injection | 2 | 3.16（2.21,4.10） | 24 | < 0.00001 |
| Two to three injections | 2 | 0.90（0.42,1.38） | 0 | 0.003 |
| 7.VAS score at 12 month |  |  |  |  |
| One injection | 1 | 2.81(1.80,3.82) | / | < 0.00001 |
| Two to three injections | 3 | 1.44(0.77,2.11) | 71 | < 0.0001 |
| 8.WOMAC score at 6 month |  |  |  |  |
| One injection | 2 | 1.16(0.42,1.90) | 38 | 0.002 |
| Two to three injections | 2 | 0.43(-0.25,1.10) | 48 | 0.21 |
| 9.WOMAC score at 12 month |  |  |  |  |
| One injection | 1 | 1.34(0.34,2.33) | / | 0.008 |
| Two to three injections | 3 | 0.84(0.07,1.61) | 80 | 0.03 |

**Supplementary table 5.** Meta-regression results.

| LogES | Coef. | Std. Err. | t | 1. |t| | [95% Conf. Interval] | |
| --- | --- | --- | --- | --- | --- | --- |
| Mesenchymal stem cell source | -2.298767 | 1.128411 | -2.04 | 0.179 | -7.153927 | 2.556392 |
| Injection frequency | -1.421876 | 0.7984355 | -1.78 | 0.217 | -4.857267 | 2.013515 |

**Supplementary table** **6**. Grade evidence profile1.

| **Outcomes** | **Illustrative comparative risks* (95% CI)** | **Quality assessment** | | | | | **No of Participants**  **(studies)** | **Quality of the evidence (GRADE)** | **Comments** |
| --- | --- | --- | --- | --- | --- | --- | --- | --- | --- |
|  |  | **Risk of**  **bias** | **Inconsistency** | **Indirectness** | **Imprecision** | **Publication**  **bias** |  |  |  |
| **VAS at 6 month** | The mean meta-analysis of VAS at 6 month in the intervention groups was  **2.00 standard deviations higher**  (0.74 to 3.62 higher) | no serious | no serious | no serious | serious | undetected | 132  (4 studies) | ⊕⊕⊕⊝  **Moderate**^1,2^ | SMD 2.00 (0.74 to 3.62) |
| **VAS at 12 month** | The mean meta-analysis of VAS at 12 month in the intervention groups was  **1.72 standard deviations higher**  (1.00 to 2.45 higher) | serious | no serious | no serious | no serious | undetected | 223  (4 studies) | ⊕⊕⊕⊝  **Moderate**^2,3^ | SMD 1.72 (1.00 to 2.45) |
| **Total WOMAC at 6 month** | The mean meta-analysis of WOMAC at 6 month in the intervention groups was **0.78 standard deviations higher**  (0.20 to 1.37 higher) | no serious | no serious | no serious | serious | undetected | 137  (4 studies) | ⊕⊕⊕⊝  **Moderate**^2^ | SMD 0.78 (0.20 to 1.37) |
| **Total WOMAC at 12 month** | The mean meta-analysis of WOMAC at 12 month in the intervention groups was **0.93 standard deviations higher** (0.28 to 1.58 higher) | serious | no serious | no serious | serious | undetected | 217  (4 studies) | ⊕⊕⊝⊝  **Low**^1,2^ | SMD 0.93 (0.28 to 1.58) |
| **WORMS** | The mean meta-analysis of WORMS in the intervention groups was **24.11 higher** (18.30 to 29.92 higher) | no serious | no serious | no serious | serious | undetected | 254  (2 studies) | ⊕⊕⊕⊝  **Moderate**^2,4^ | SMD 24.11 (18.30 to 29.92) |
| **MOCART** | The mean MOCART in the intervention groups was **11.82 higher** (7.86 to 15.78 higher) | no serious | no serious | serious | serious | undetected | 127  (2 studies) | ⊕⊕⊝⊝  **Low**^2,3,,4^ | SMD 11.82 (7.86 to 15.78) |

GRADE Working Group grades of evidence: **High quality:** Further research is very unlikely to change our confidence in the estimate of effect. **Moderate quality:** Further research is likely to have an important impact on our confidence in the estimate of effect and may change the estimate.**Low quality:** Further research is very likely to have an important impact on our confidence in the estimate of effect and is likely to change the estimate.**Very low quality:** We are very uncertain about the estimate.

1.High heterogeneity (I^2^> 50%) was found.

2.All meta-analysis had sample sizes of no more than 400 patients.

3.Some of the included studies did not blind the participants.

4. Some studies were not statistically significant.

**Supplementary table** **7** . Grade evidence profile2.

| **Outcomes** | **Illustrative comparative risks* (95% CI)** | | **Quality assessment** | | | | | **Relative effect**  **(95% CI)** | **No of Participants**  **(studies)** | **Quality of the evidence**  **(GRADE)** |
| --- | --- | --- | --- | --- | --- | --- | --- | --- | --- | --- |
|  | **Control** | **Treatment** | **Risk of**  **bias** | **Inconsistency** | **Indirectness** | **Imprecision** | **Publication**  **bias** |  |  |  |
| **Knee pain or swelling**  **events** | **Study population** | | no serious | no serious | no serious | serious | no serious | RR 1.04  (0.82 to 1.31) | 407  (7 studies) | ⊕⊕⊕⊝  **Moderate**^1^ |
|  | 278 per 1000 | 289 per 1000 |  |  |  |  |  |  |  |  |
|  | **Moderate** |  |  |  |  |  |  |  |  |  |
|  | 375 per 1000 | 390 per 1000 |  |  |  |  |  |  |  |  |

GRADE Working Group grades of evidence: **High quality:** Further research is very unlikely to change our confidence in the estimate of effect. **Moderate quality:** Further research is likely to have an important impact on our confidence in the estimate of effect and may change the estimate.**Low quality:** Further research is very likely to have an important impact on our confidence in the estimate of effect and is likely to change the estimate.**Very low quality:** We are very uncertain about the estimate.

1.All meta-analysis had sample sizes of no more than 400 patient
