# Supplementary figures and images for: Effect of intra-knee injection of autologous adipose stem cells or mesenchymal vascular components on short-term outcomes in patients with knee osteoarthritis: an updated meta-analysis of randomized controlled trials
Source: Arthritis Res Ther. 2023 Aug 10;25:147. doi: 10.1186/s13075-023-03134-3 (PMC10413774; doi:10.1186/s13075-023-03134-3)

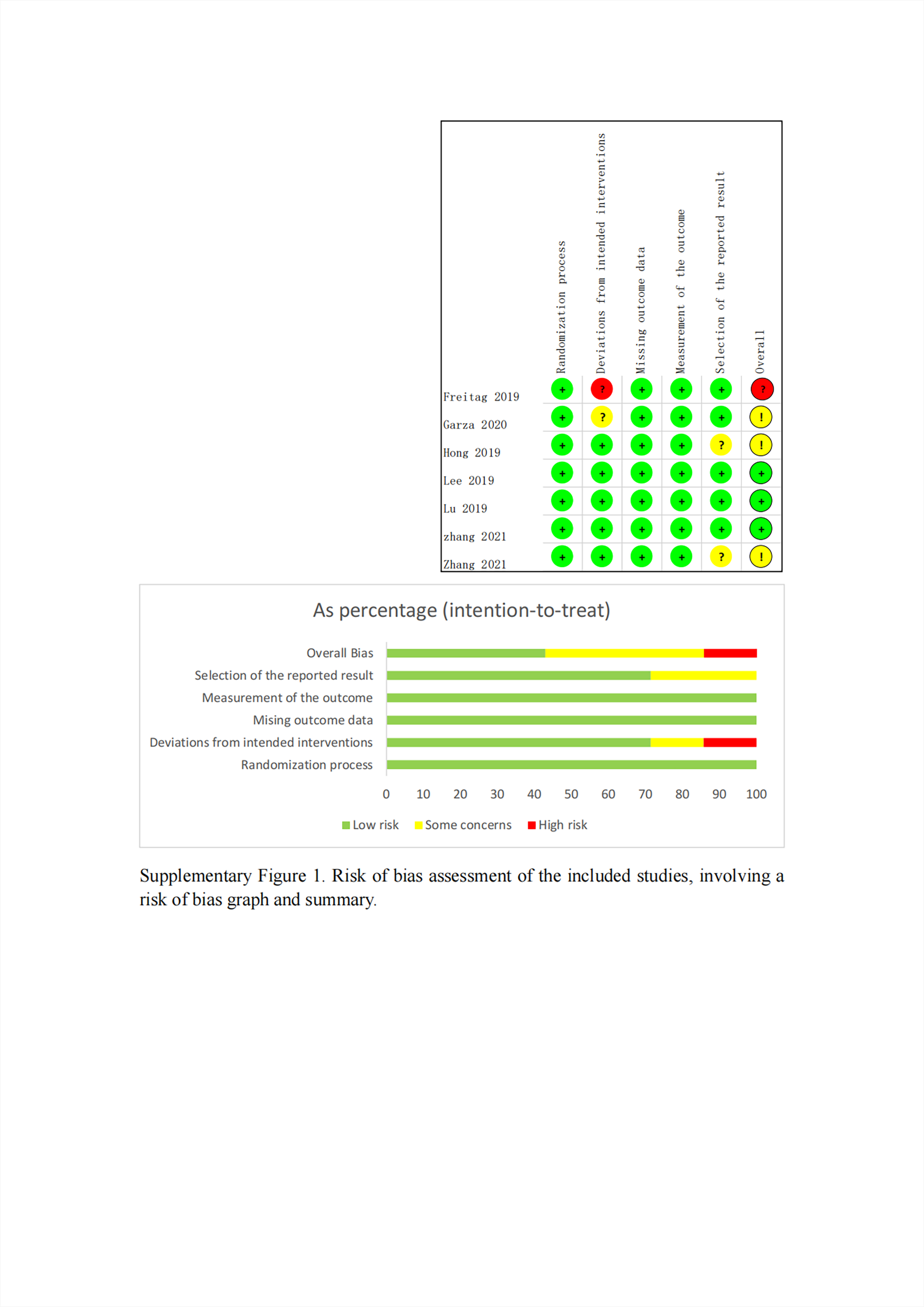

Supplement: Supplementary file 2 — Additional file 2: Supplemental Fig. 1. Risk of bias assessment of the included studies, involving a risk of bias graph and summary. [file 13075_2023_3134_MOESM2_ESM.tif]
